# Evaluation of mental functioning of patients with rheumatoid arthritis

**DOI:** 10.1192/j.eurpsy.2023.932

**Published:** 2023-07-19

**Authors:** I. Mnif, A. Feki, I. Sellami, Z. Gassara, S. Ben Djemaa, M. Ezzeddine, M. H. Kallel, H. Fourati, S. Baklouti

**Affiliations:** 1 Rheumatology; 2 occupational medecine, Hedi Chaker Hospital, university of Sfax, Sfax, Tunisia

## Abstract

**Introduction:**

Rheumatoid arthritis (RA) is a chronic inflammatory degenerative disease whose symptoms are mainly joint with significant functional impact, resulting in a restriction of the activities of the patient and increasing the impact on mental well-being.

**Objectives:**

This study aimed to screen for mental functioning in RA patients, detect anxiety and depression, evaluate self-esteem and study its relation to clinical parameters, as well as disease activity.

**Methods:**

Single-centre cross-sectional study, involving patients with RA using the hospital anxiety and depression scale (HADs). Rosenberg scale was used to evaluate self-esteem. We evaluated the RA severity Disease Activity Severity (DAS 28).

**Results:**

Fifty patients were included. The average age was 54 years [24-72]. The mean duration of the disease was 10 years. Thirty-nine patients had immunopositive RA with a mean Rheumatoid Factor level of 189.1 ± 291.3 U/ml and a mean anti-CCP antibody level of 165 ± 225.3 U/ml. At diagnosis, the mean DAS 28 of the patients was 5.1 ± 1.4. Moderately active and highly active RA were predominant with percentages of 40% and 50% respectively. All patients were treated, and 36% received biological treatment.

Depression was noted in 42% of the patients with a mean score of 10.1 ± 3.7. Anxiety was noted in 50% of the patients with a mean score of 10.3 ± 4.

In this study, we did not find a statistically significant association between disease activity and depression or anxiety scores (p=0.6 and p=0.1 respectively).

The mean Rosenberg scale score was 27± 3. Sixty-eight per cent of patients had low self-esteem, twenty-one per cent had moderate self-esteem and eleven per cent very low self-esteem. Disease activity was associated with low self-esteem.

**Conclusions:**

RA is a chronic inflammatory disease that has a significant impact on the mental health and quality of life of patients. The detection and treatment of psychiatric disorders such as anxiety, depression and low self-esteem; improve the care of patients with RA.

**Disclosure of Interest:**

None Declared

